# Neurorehabilitation technologies and functional recovery after brain injury: influence of sex, an integrative review

**DOI:** 10.3389/fdgth.2025.1677873

**Published:** 2026-01-23

**Authors:** Phoebe Bennett, Neil Barr

**Affiliations:** 1 DeGroote School of Business, McMaster University, Hamilton, ON, Canada; 2 Digital Solutions, St Joseph’s Healthcare Hamilton, Hamilton, ON, Canada

**Keywords:** brain computer interface, functional recovery, neurorehabilitation after brain injury, robotics, sex-difference, stroke, traumatic brain injury, virtual reality

## Abstract

**Background:**

Acquired brain injury (ABI), which includes traumatic brain injury (TBI) and stroke, is a leading cause of disability. Evidence shows that sex may influence functional recovery post-acquired brain injury, potentially due to biological (e.g., hormones) and social factors (e.g., caregiver availability). Meanwhile, new neurorehabilitation technologies—such as virtual reality, robotic-assistance, and brain-computer interfaces—offer promising avenues for improving functional outcomes. Understanding how these technologies interact with sex differences could advance equitable and personalized healthcare.

**Research question:**

Does evidence support a rationale for studying, developing, or employing neurorehabilitation technologies differently in males and females to improve functional outcomes post-ABI?

**Methodology:**

An empirical integrative narrative review was conducted. Searches were performed in PubMed, Cochrane Library, and OVID, focusing on adult populations with ABI. Key terms encompassed “acquired brain injury,” “sex differences,” and “neurorehabilitation technologies.” Fifty-nine studies met inclusion criteria, spanning diverse methodologies, settings, and cultural contexts. Data were synthesized to compare functional outcomes impacted by sex and by neurorehabilitation technologies.

**Results:**

Findings indicate that the effect of sex on neurorehabilitation outcomes is multifaceted. Studies using functional independence measures often reported no significant sex differences, whereas more specific measures (e.g., those measuring cognitive or social functions) identified notable sex effects. Neurorehabilitation technologies showed positive outcomes in various functional domains (e.g., upper extremity motor function, gait, cognition), but most studies focused on stroke.

**Discussion:**

Current research does not support the use of sex-differentiated technology interventions to target upper extremity motor function or global functional independence post-stroke. Sex-differentiated treatment may be relevant for other functional domains such as cognitive recovery, psychological well-being and social outcomes, but this requires further research, particularly for non-stroke ABI.

**Conclusion:**

These findings suggest that some neurorehabilitation technologies can be applied without sex-specific modification, whereas others may benefit from sex-specific considerations. Owing to methodological limitations and sparse data, especially for TBI, additional investigations are warranted. As novel neurorehabilitation technologies evolve, accounting for sex differences may enhance personalized care and optimize long-term outcomes.

## Introduction

Acquired brain injury (ABI) affects a large percentage of the Canadian population and is a leading cause of disability across the nation (1–3). ABI is defined as any injury to the brain that occurs after birth and is not the result of a disease, or genetic or developmental disorder (2). Complete functional recovery post-ABI is uncommon and can be defined as the total return to previous activity levels following injury, or the absence of disability initially caused by the injury (1, 2, 4). There are specific types of functional recovery related to the symptoms of ABI (5). Two major types include cognitive functional recovery, which refers to the return of the pre-injury ability levels to complete cognitive functions such as speech, language processing, attention, visual-spatial processing, and memory; and motor functional recovery, which refers to the return of pre-injury ability levels to perform voluntary movements and postures (5). Traumatic Brain Injury (TBI) and stroke are two common types of ABI (1, 2). Although TBI and stroke result from different primary insults, their underlying pathophysiologies are similar (i.e., neuronal cell death through loss of cerebral blood flow (6). Additionally, similar post-acute rehabilitation methods are used for both injuries and research has shown comparable recovery patterns for stroke and TBI (7, 8).

### Sex differences in functional recovery

Sex differences in functional recovery post-ABI, although dependent on the type of ABI, the age of the individual, and the functional outcome measured, are frequently observed (9–13). It has been postulated that differences in functional recovery between the sexes following ABI are due to both biological and social factors (11, 14). Sex is often conceptualized as biological characteristics derived from sex-chromosomes, whereas gender refers to social and personal constructs historically associated with biological sex; for the purposes of this review, both social and biological factors are considered in the definition of sex.

Differences in brain structure and hormones between the sexes play a role in functional recovery (9, 14, 15). For example, estrogen has been shown to provide neuroprotection, improving females' recovery from TBI by reducing lesion size, edema, and cell death, and improving neuronal cell count; however, these protective effects are diminished post-menopause making age an important consideration (15–17). Bonkhoff et al. found sex-specific lesion patterns in patients with ischemic stroke, which they proposed may lead to differences in functional outcomes. Specifically, female patients were found more likely to experience certain lesion patterns in the left-brain hemisphere that are linked to severe stroke and worsened cognitive functional outcomes. In terms of social factors, the age at which males typically experience stroke is linked to improved caregiver availability, and males are less likely to be single post-stroke, although these factors have not been systematically studied (11, 12, 18).

Despite extensive study of sex differences in ABI, there is no definitive answer as to which sex experiences worse outcomes post-ABI, and studies continue to find no sex-related significant difference in functional recovery. For example, a prospective multicenter cohort study by Yun et al. (13) found persistent sex differences in functional recovery patterns after ischemic stroke up to 60 months post-injury, as measured by 5 different functional outcome scales. However, another retrospective cohort study of similar proportions found no difference in functional outcomes for severe stroke, although only motor outcomes were measured (106).

Findings on sex differences in functional recovery post-ABI are impacted by numerous confounding factors, likely contributing to their inconsistency. As the comparison of Yun et al. (13) and Scrutinio et al. (106) demonstrates, the wide variety of outcome measures used to assess functional recovery make it difficult to appropriately conclude the effect of sex. Cross-study comparison of individual functional outcomes may be beneficial, and the findings of Gupte et al. (9) suggest this approach; their systematic review of sex differences in TBI found that females’ improved performance is inconsistent across functional outcomes, highlighting measure variability. Amid mixed findings on the factors impacting functional recovery post-ABI, it is important to further consider interventions aimed at improving functional recovery, such as neurorehabilitation.

### Neurorehabilitation

Neurorehabilitation involves different interventions when rehabilitating cognitive vs. motor functions (19). Cognitive rehabilitation is of considerable interest following ABI due to the phenomenon of neuroplasticity; particularly the opportunity for modulation of synaptic transmission, synaptogenesis and neurogenesis, and significant evidence of cortical reorganization following brain injury, regardless of age (20). Although cognitive rehabilitation is recommended by many clinical practice guidelines for rehabilitation of ABI, benefits may be short-term and have minimal translation into everyday functioning (21). Evidence to support the lasting efficacy of motor function interventions is also limited and complete motor functional recovery is unlikely regardless of the neurorehabilitation received (22, 107). That said, multiple studies have proposed motor functional recovery can continue far past the post-acute stage of rehabilitation (e.g., 7 years post-injury) due to neuroplasticity (23, 24).

The limited efficacy of typical neurorehabilitation in improving functional outcomes as well as the cost implications of ABI-related disabilities has led to an increased interest in research that examines the efficacy of new technologies at improving functional recovery post-ABI (6, 25).

### Neurorehabilitation technologies

Examples of technologies being newly explored to support functional recovery post-ABI include virtual reality (VR), brain computer interfaces (BCI), robotic-assistance devices, and electronic brain stimulation (26). Multiple studies have found these technologies to be efficacious at improving functional recovery post-ABI in combination or in comparison with typical neurorehabilitation interventions (25, 27). For example, De Luca et al. (28); De Luca et al. (29) found virtual rehabilitation to be a promising intervention for improving executive functions post-right hemisphere stroke, in comparison with typical cognitive rehabilitation alone. Additionally, with the use of Robotic Verticalization Training (RVT), De Luca et al. (30) found improvements in functional outcomes for individuals in a chronic Minimally Conscious State (MCS) caused by ABI. Lastly, Brain-Computer Interface (BCI) therapy has been shown to improve upper extremity motor function for individuals with stroke of various severities, regardless of injury stage (31).

### Aims & purpose

Due to the suggested influence that sex has on functional recovery post-ABI, and the promising potential of new neurorehabilitation technologies to improve functional recovery, an empirical integrative narrative review was conducted to explore how sex influences the efficacy of neurorehabilitation technologies at improving functional recovery post-ABI. The main objective of this review was to establish a basis for whether sex should be considered when studying, developing or employing new neurorehabilitation technologies. In a world that is becoming increasingly reliant on technology, this review aimed to contribute to a growing body of research on implementing equitable and personalized healthcare technology.

Despite extensive research on sex differences or emerging neurorehabilitation technologies, it remains unclear whether rehabilitation technology should be sex-differentiated. In alignment with Health Canada's Sex- and Gender- Based Analysis Plus (SGBA+) action plan, this study addresses that gap by examining how sex might influence technology efficacy as an intervention for ABI (32). To this authors knowledge, this is a novel approach given that no prior review has explicitly aligned with SGBA + to understand the influence of sex on neurorehabilitation technologies.

## Methods

To study the influence of sex on the efficacy of neurorehabilitation technologies post-ABI, an empirical integrative narrative review was selected to enable synthesis of heterogenous domains of neurorehabilitation technology (e.g., Brain Computers Interfaces, Virtual Reality and Robotics). These domains vary widely in study methodology and are extremely limited in reporting of sex-specific data, precluding uniform inclusion criteria necessary for systematic review. Although several of the domains of interest have substantial bodies of literature (e.g., VR in post-stroke rehabilitation), these domains have been studied in isolation rather than in relation to sex as a moderating variable. Therefore, a narrative approach allows for alignment with SGBA + by permitting synthesis of evidence across studies stratifying functional outcomes by sex, and those that focus on neurorehabilitation technologies.

The review was conducted per the narrative review guidelines from Green et al. (5) and drew on evidence-based studies with diverse methodologies to summarize and interpret current knowledge, identify patterns, and highlight gaps in the literature (33). Key information (injury type, intervention, outcomes targeted, efficacy, and whether sex differences were found) was charted from selected studies and tabulated (see Tables 1, 2). Functional outcomes outlined in Tables 1, 2 were organized into a Venn diagram to conceptualize the overlap of outcomes impacted by sex with those influenced by neurorehabilitation technologies (see Figure 2).

**Table 1:** Human studies showing influence of Sex on functional outcomes post-ABI.

References	ABI Type	Functional Outcome	Measurement Tool(s)^a^	Sex Differences (Worse Outcome)
(34)	TBI (Mild to Severe)	Depression	Structured Clinical Interview for DSM-IV diagnosis	Yes (Female)
(35)	TBI (Moderate to severe)	Quality of Life (Mental Health)	Medical Outcome Short Form 36	Yes (Female)
(36)	TBI (Moderate to severe)	Sexual Function	DISF-SR, GSSI	Yes (Female)
(37)	TBI (Moderate to severe)	High Level Motor Function	HiMAT	Yes (Female)
(38)	TBI (Mild to Severe)	Depression	HADS-D	Yes (Female)
(39)	TBI (Severe)	Empathy	Interpersonal Reactivity Index Empathic Concern (EC) and Perspective Taking (PT) subscales	Yes (Female)
(40)	TBI (Mild to Severe)	Visual memory	CANTAB	Yes (Male)
(41)	TBI (Mild to Severe)	Functional Independence	GOS-E	No (N/A)
(42)	TBI (Moderate to severe)	Emotion Recognition/ Social Impairment	KDEF, ERT	Yes (Male)
(43)	TBI (Moderate to severe)	Headaches, Dizziness, Noise Sensitivity, Sleep Disturbances, Daily Functioning	PCL, HIFI	Yes (Mixed Results)
(44)	Stroke	Disability	iADL	Yes (Female)
(110)	TBI (Moderate to severe)	Injury-related Deficit Awareness	PCRS and FrSBe	Yes (Male)
(45)	Stroke	Upper Extremity Motor Function	Purdue Pegboard Assessment	Yes (Male Age <62)
(46)	TBI (Severe)	Cognitive Function	FIM Cognitive	Yes (Male)
(13)	Stroke	Motor Function, Ambulatory Function, Cognitive Function, Language function, Swallowing, Active Daily Living	FMA, FAC, K-MMSE, Short K-FAST, K-MBI	Yes (Female)
(47)	Stroke	Disability	mRS	Yes (Mixed Results)
(48)	Stroke	Disability	mRS	Yes (Mixed Results)
(49)	Stroke	Disability after intravenous thrombolysis	mRS	Yes (Female)
(50)	Stroke	Functional Independence	FIM	No (N/A)
(51)	TBI	Attention, Working memory, Visual Analytical skills	WAIS,TMT-A, TMT-B, Symbol Digit Modalities Test, Wechsler Memory Scale Logical Memory, Rey Auditory Verbal Learning Test, Token Test, Controlled Oral Word Association Test, Benton Visual Discrimination Test, WAIS Block Design, Wisconsin Card Sorting Test, Grooved Pegboard Test	Yes (Mixed Results)
(52)	Stroke	Functional Independence	FIM	No (N/A)
(53)	TBI	Functional Independence	GOS	No (N/A)
(54)	Stroke	ADLs, Physical Function, Thinking, Language, Energy	Barthel Index SS-QoL	Yes (Female)
(55)	Stroke	Functional Independence	FIM	Yes (Female)
(56)	Stroke	Cognitive Function, Functional Independence, Physical Function & Activity, Strength, Memory, Emotion, Communication, iADLS, Mobility, Hand function, Social Participation	MMSE, NIHSS, Barthel Index, Modified Rankin Scale, Stroke Impact Scale,	Yes (Female)
(57)	Stroke	Functional Independence	FIM	No (N/A)
(58)	Stroke (Mild to Moderate)	Daytime sleepiness	ESS, PSQI, HADS	Yes (Mixed Results)
(59)	Stroke	Functional Independence	mRS, NIHSS	No (N/A)
(60)	Stroke	Dependency	NIHSS, mRS, GCS	Yes (Female, 3 months post-injury)
(61)	Stroke	Disability	mRS, NIHSS	No (N/A)
(62)	Stroke	Motor Performance, Activity and Participation	FMS, FIM-Motor Sub-scale, London Handicap Scale	No (N/A)
(16, 17)	Stroke	Disability	RS	Yes (Male)
(63)	Stroke	Functional Outcome	NIHSS, RS	No (N/A)
(64)	Stroke	Upper Extremity Motor Function	SIS-H, FMUE,	No (N/A)
(65)	Stroke	Disability	mRS	Yes (Female)
(66)	Stroke	Quality of Life, Mental Health, Social Satisfaction, Emotional Problems, Physical Health, Physical Activities, Pain, Fatigue, General Health, Social Activity, Cognitive Status, Cognitive Changes, Disability	BI, mRS, PROMs-10, TICS, IQCODE	Yes (Female)
(67)	Stroke	Quality of Life (physical and psychological), Memory, Anxiety	HADS, WHOQoL-Bref	Yes (Female)
(68)	TBI (Moderate to severe)	Sleep Wake Disturbances, Verbal Learning, Quality of Life, Depression, Sensory Function, Motor Function, Cognitive Function	MPAI, CVLT, Neuro QoL, BDI	Yes (Female)

a BDI, Beck Depression Inventory; BI, Barthel Index; CANTAB, Cambridge Neuropsychological Test Automated Battery; COWAT, Controlled Oral Word Association Test; CVLT, California Verbal Learning Test; DISF-SR, Dysexecutive Syndrome Questionnaire Self-Report; ERT, Emotion Recognition Task; ESS, Epworth Sleepiness Scale; FAC, Functional Ambulation Category; FIM, Functional Independence Measure; FMA, Fugl-Meyer Assessment; FMA-UE, Fugl-Meyer Upper Extremity Assessment; FMS, Functional Mobility Scale; FrSBe, Frontal Systems Behavior Scale; GCS, Glasgow Coma Scale; GOS, Glasgow Outcome Scale; GOS-E, Glasgow Outcome Scale—Extended; GSSI, Global Severity of Symptoms Index; HADS, Hospital Anxiety and Depression Scale; HADS-D, Hospital Anxiety and Depression Scale—Depression Subscale; HIFI, Head Injury Family Interview; HiMAT, High-Level Mobility Assessment Tool; IADL, Instrumental Activities of Daily Living; IQCODE, Informant Questionnaire on Cognitive Decline in the Elderly; IRI-EC, Interpersonal Reactivity Index—Empathic Concern Subscale; IRI-PT, Interpersonal Reactivity Index—Perspective Taking Subscale; KDEF, Karolinska Directed Emotional Faces; K-MBI, Korean Modified Barthel Index; K-MMSE, Korean Mini-Mental State Examination; LHS, London Handicap Scale; MMSE, Mini-Mental State Examination; MPAI, Mayo-Portland Adaptability Inventory; mRS, Modified Rankin Scale; Neuro-QoL, Quality of Life in Neurological Disorders Measure; NIHSS, National Institutes of Health Stroke Scale; PCL, PTSD Checklist; PCRS, Patient Competency Rating Scale; PROMs-10, Patient Reported Outcome Measures-10; PSQI, Pittsburgh Sleep Quality Index; RAVLT, Rey Auditory Verbal Learning Test; RS, Rankin Scale; SCID, Structured Clinical Interview for DSM-IV Diagnosis; SDMT, Symbol Digit Modalities Test; SF-36, Medical Outcome Short Form 36; Short K-FAST, Short Korean version of the Frenchay Aphasia Screening Test; SIS, Stroke Impact Scale; SIS-H, Stroke Impact Scale—Hand Subscale; SS-QoL, Stroke Specific Quality of Life Scale; TICS, Telephone Interview for Cognitive Status; TMT-A, Trail Making Test Part A; TMT-B, Trail Making Test Part B; WAIS, Wechsler Adult Intelligence Scale; WCST, Wisconsin Card Sorting Test; WHOQoL-BREF, World Health Organization Quality of Life—Brief; WMS, Wechsler Memory Scale.

**Table 2:** Human studies showing influence of neurorehabilitation technologies on functional outcomes post-ABI*.*

References	ABI Type	Technology	Functional Outcome	Measurement Tool(s)^b^	Significant Effect?
(69)	Stroke	Virtual Reality, Augmented Reality, Mixed Reality	Upper Extremity Motor Function, ADLs	FMA-UE, FIM, BBT, WMFT	Yes (Not Upper Extremity Function as measured by BBT, WMFT)
(70)	Stroke	Virtual Reality	Global cognition, Attention, Memory, Language	MoCA, K-MoCA, K-MMSE, MMSE, TMT-A, Forward Digit Span Test, Stroke Impact Scale-Memory Domain, Stroke Impact Scale-Communication Domain, Neurobehavioral Functioning Inventory-Communication Domain	No
(71)	Stroke	Virtual Reality	Upper- and lower-extremity motor function, balance, gait, ADLs	FMA-UE, Action Research Arm Test, Wolf Motor Function Test, FMA-LE, FAC, BBS, Time Up and Go, Velocity, Cadence, Modified BI, FIM, BBT, 10MWT, Auditory Continuous Performance Test, MMSE, Visual Continuous Performance Test	Yes (Not cognition as measured by Auditory Continuous Performance Test, MMSE, Visual Continuous Performance Test)
(72)	Stroke	Robot-Assisted Gait Training	Balance, functional gait (endurance, stability)	10MWT, 6MWT, FIM, MI, FAC, Tinetti Test	Yes
(73)	Stroke	Upper Extremity Exoskeleton and Virtual Reality	Self-Care, Upper Extremity Motor Function, Attention, Memory, Visuospatial abilities, Complex commands, Anxiety Level	FIM, FMA-UE, MAS, HTT, BBT, ROM, MMSE, Addenbrooke’s Cognitive Examination-Revised, HAD	Yes
(74)	Stroke	Robot-Assisted Training	Upper Extremity Motor Function, ADLs	FMA-UE, Modified BI, MAS, FIM, WMFT	Yes (Not MAS, FIM, WMFT)
(75)	Stroke	Robot-Assisted Task-Oriented Training	Upper Extremity Motor Function, ADLs	FMA-UE, BBT, EDC, grip dynamometer, Semmes-Weinstein hand monofilament, Revised Nottingham Sensory Assessment, MBI	Yes
(76)	Stroke	Robot-Assisted Training	Upper Extremity Motor Function	ARAT	No
(77)	Stroke with Ataxia	Robot-Assisted Gait Training	Balance, Gait, Fine Motor Skills, Functional Independence	BBS, TUG, FIM, SARA	Yes (Equal to therapist-assisted gait training)
(78)	Stroke	Robot Assisted Therapy and Mirror Therapy	Motor Function, ADLs, Self-Efficacy,	FMA, WMFT, Nottingham Extended ADL Scale, Stroke Self-Efficacy Questionnaires, DL-SES	Yes (Only Self-Efficacy)
(79)	Stroke	Lower Extremity Exoskeleton treadmill training	Lower-Extremity Motor Function, Balance	FACS, BBS, 10MWT, 6MWT	Yes (Only Balance)
(80)	Stroke	Overground Robot-Assisted Gait Training	Gastrointestinal function, Psychological well-being (Mood, Coping Strategies, Social Support), Quality of Life	CONST, PGWBI, FIM, MoCA, HRS-D, Cope-NIV, SF-12, 10MWT, TUG, RMI	Yes
(81)	TBI	Lower Extremity Exoskeleton treadmill training and Virtual Reality	Mood, Perceived Physical Well-being, Global cognitive function, Executive functions (preservation, planning, classification), cognitive flexibility and shifting skills, selective attention, Quality of life (perceived mental and physical state)	BDI-II, SF-PH, LPG, MoCA, FAB, WEIGL, TMT-A, TMT-B, SF12 TOT, SF12-MH; SF-PH	Yes
(82)	Stroke	Lower Extremity Exoskeleton treadmill training	Lower Extremity Motor Function, Balance, Functional Independence, Psychological well-being (Depression, Social Support, Voidance Strategies, Positive Attitude, Problem Solving, Turning to Religion)	MAS, FIM, Tinetti test, HRS-D, COPE, PGWBI	Yes
(83)	Stroke	Brain Computer Interface Training	Upper Extremity Motor Function	FMA	Yes
(84)	Quadriplegia^a^	Brain/Neural Hand Exoskeleton	Functional Independence, Upper Extremity Motor Function	SCIM, FIM	Yes
(85)	Stroke	Brain Computer Interface-monitored Motor Imagery	Functional Independence, Upper Extremity Motor Function	FMA-UE, EEG	Yes
(86)	Stroke	Brain Computer Interface Training	Upper Extremity Motor Function	cFMA, EMG, fMRI	Yes
(87)	Stroke (Case Study)	Virtual Reality	Upper Extremity Motor Function	TULIA	Yes
(88)	Stroke (Case Study)	Brain Computer Interface Driven Virtual Reality	Upper Extremity Motor Function	FMA-UE, MoCa, MAS, SIS, fMRI	Yes (Only motor function as measured by FMA-UE)
(28, 29)	TBI	Virtual Reality	Cognitive Function, cognitive flexibility, attentional shifting, visual search, executive functions, visuospatial functions	MoCa	Yes

a Quadriplegia is not considered an ABI, rather a consequence of brainstem stroke or other conditions such as Spinal Cord Injury (SCI); because hemiplegia is a common symptom of ABI, a study on individuals with quadriplegia was included.

b 10MWT, 10-Meter Walk Test; 10mWT, 10-meter Walk Test; 6MWT, 6-Minute Walk Test; 6MWT, Six-Minute Walk Test; ACE-R, Addenbrooke’s Cognitive Examination-Revised; ACPT, Auditory Continuous Performance Test; ARAT, Action Research Arm Test; BBS, Berg Balance Scale; BBT, Box and Block Test; BDI-II, Beck Depression Inventory-II; BI, Barthel Index; cFMA, Computerized Fugl-Meyer Assessment; cFMA, Computerized Fugl-Meyer Assessment; CONST, Constipation Assessment Scale; CONST, Constipation Scoring System; COPE-NIV/COPE, Coping Orientation to Problems Experienced (Inventory); DL-SES, Daily Living Self-Efficacy Scale; DL-SES, Daily Living Self-Efficacy Scale; EDC, Extensor Digitorum Communis (muscle assessment); EEG, Electroencephalography; EMG, Electromyography; FAB, Frontal Assessment Battery; FAC, Functional Ambulation Category; FACS, Functional Ambulation Classification Scale; FIM, Functional Independence Measure; FMA, Fugl-Meyer Assessment; FMA-LE, Fugl-Meyer Assessment—Lower Extremity; FMA-UE, Fugl-Meyer Assessment—Upper Extremity; fMRI, Functional Magnetic Resonance Imaging; HAD, Hospital Anxiety and Depression Scale; HRS-D, Hamilton Rating Scale for Depression; HTT, Hand Tapping Test; IQCODE, Informant Questionnaire on Cognitive Decline in the Elderly; K-MMSE, Korean Mini-Mental State Examination; K-MoCA, Korean Montreal Cognitive Assessment; LPG, Line-Pegging Test; MAS, Modified Ashworth Scale; MBI, Modified Barthel Index; MI, Motricity Index; MI, Motricity Index; MMSE, Mini-Mental State Examination; MoCA, Montreal Cognitive Assessment; MoCA, Montreal Cognitive Assessment; NEADL, Nottingham Extended Activities of Daily Living Scale; NFI, Neurobehavioral Functioning Inventory; NSA (Revised), Revised Nottingham Sensory Assessment; PGWBI, Psychological General Well-Being Index; RMI, Rivermead Mobility Index; ROM, Range of Motion; SARA, Scale for the Assessment and Rating of Ataxia; SCIM, Spinal Cord Independence Measure; SF-12, 12-Item Short Form Health Survey; SF12-MH, Short Form 12—Mental Health Subscale; SF12-PH/SF-PH, Short Form 12—Physical Health Subscale; SIS, Stroke Impact Scale; Stroke SEQ, Stroke Self-Efficacy Questionnaire; Tinetti Test, Tinetti Performance-Oriented Mobility Assessment; TMT-A, Trail Making Test Part A; TMT-B, Trail Making Test Part B; TOT CONST, Total Constipation Score; TUG, Timed Up and Go Test; TULIA, Test of Upper Limb Apraxia; V-CPT, Visual Continuous Performance Test; WEADL, Nottingham Extended Activities of Daily Living Scale; WMFT, Wolf Motor Function Test.

### Saturation and reviewer procedures

Per Sukhera, this narrative review did not aim to include all relevant literature related to neurorehabilitation technologies and sex differences post-ABI. For studies examining sex differences, given their abundance, saturation was defined conceptually rather than quantitatively. Aligning with Glaser and Strauss’ (89) grounded theory, the single non-blinded reviewer determined that sufficient analyses were achieved once new articles failed to introduce new conceptual categories relevant to the impact of sex on functional outcomes. Similarly, review of the impact of VR and robotics on functional outcomes post-stroke was considered complete once continuous search revealed no additional studies whose findings added diversity to the sample. For scarcer study themes (e.g., studies on BCI and studies on neurorehabilitation technologies impacting TBI), all related studies that explicitly stated impacts on functional outcomes were included.

### Sources & search strategy

To gather relevant literature, a search for evidence-based studies was conducted using multiple platforms including:

- PubMed
- Cochrane Library
- OVID Databases

The search incorporated a combination of Medical Subject Headings (MeSH) and keywords, including terms related to:

- **Population**: Acquired brain injury (ABI), traumatic brain injury (TBI), stroke, cerebral infarction, moderate to severe brain injury, or brain damage.
- **Concept**: Neurorehabilitation technologies, neurorehabilitation, virtual reality (VR), robotics, brain-computer interfaces (BCI), robotics, or rehabilitation technology.
- **Context**: Functional recovery, motor functional outcomes, cognitive functional outcomes, motor recovery, cognitive recovery, psychological recovery, sex differences, or gender differences.

The search strategy used Boolean operators (e.g., AND/OR) to combine terms flexibly to ensure comprehensive coverage (e.g., Acquired brain injury AND sex differences OR gender differences AND functional recovery). Search by hand was conducted to supplement knowledge of themes with scarcer evidence.

### Eligibility criteria & quality assessment

Studies were eligible for inclusion if they met the following criteria:

- **Population:** Adults (≥18 years) recovering from acquired brain injury, specifically stroke or traumatic brain injury due to these injuries’ comparability.
- **Intervention/Domain:** Research discussing target neurorehabilitation technologies (e.g., virtual reality, robotics, brain-computer interfaces, telerehabilitation) and their influence on functional outcomes
- **Sex Relevance:** Studies that reported, analyzed, or discussed sex- or gender-related difference, or provided data from which such differences could be inferred; this review did not exclude studies which conflated or combined the conceptual definitions of sex and gender.
- **Publication characteristics:** Peer-reviewed articles published within the past 20 years, with full text available in English. Grey literature, editorials and opinion pieces were reviewed for introduction to supportive literature but not considered in the results.

Studies were excluded if they:

- Examined only mortality as an outcome measure.
- Used animal models of acquired brain injury.
- Focused on pediatric populations.
- Evaluated neurorehabilitation technologies out of study scope.
- Evaluated ABI-types other than stroke or TBI.

Study quality was assessed throughout review by examining factors such as methodological transparency and clarity of outcome measures when interpreting findings. An appraisal tool was not used given that the review draws from a multitude of different study designs preventing use of a single validated appraisal tool; Applying multiple appraisal tools would have caused fragmented analysis and prevented clean comparison across domains.

## Results

Criterion for sufficient saturation of results, as defined by Glauser and Strauss' (89) concept of theoretical saturation and study availability, was met after 59 studies were reviewed. Studies found were inclusive of adult participants who had experienced an ABI or experienced symptoms consistent with brain injury, and samples ranged in size from over 2,000 to 1 individual. Data collection methods varied from randomized controlled trials to reviews with meta-analyses, to case studies. The studies reviewed took place in various geographical and cultural contexts, notably Korea, China, Europe, and North America.

### Key findings

#### Impact of sex on functional outcomes

**Figure 1:**
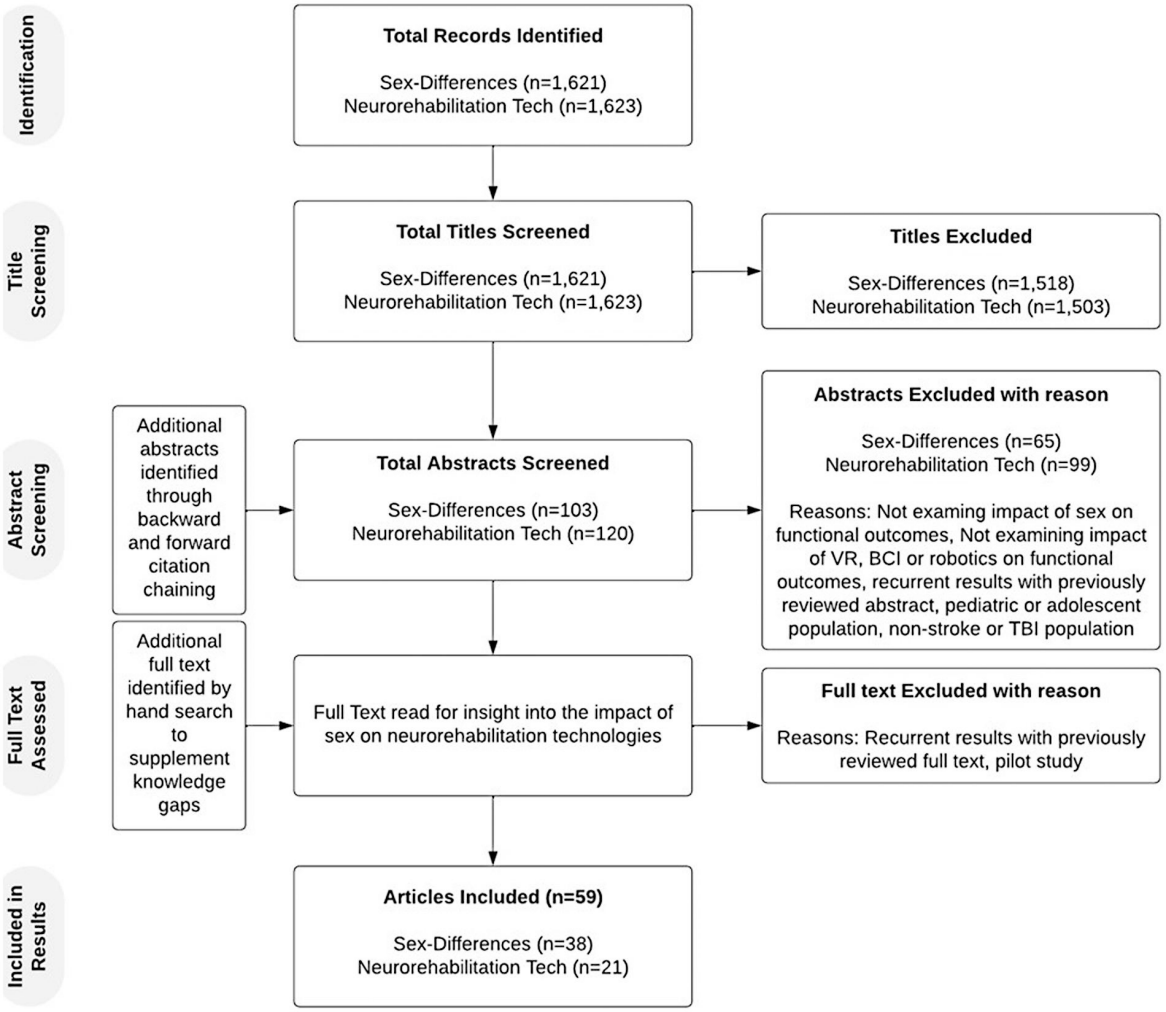
Study inclusion process.

As outlined in Figure 1, an initial search using the defined terms related to sex-differences in functional outcome post-ABI returned 1,621 sources. 103 studies were analyzed beyond their title. Studies that explicitly stated “Sex-differences” or “Gender differences” and either “traumatic brain injury” or “stroke” or an analogous term in the title were prioritized for more thorough review. The 38 studies that met eligibility criteria are included in Table 1.

The studies were evenly distributed across the types of injury (i.e., TBI and Stroke). They used a wide variety of functional outcome measures, the most common being Functional Independence Measure (FIM), Global Outcome Scale (GOS) (or GOS-Extended) and Modified Rankin Scale (mRS), to assess functional independence or global disability. A few of the studies specified the type of stroke studied but most were non-specific. Alternatively, almost all TBI studies specified what severity of TBI was being studied. Of the 38 studies, several concluded that females had worse outcomes post-ABI (*n* = 17), about a third of these concluded males had worse outcomes (*n* = 6), and a few concluded that sex-differences existed with mixed results (*n* = 5) or that there were no sex-differences at all (*n* = 10). Of the studies which examined motor outcomes (*n* = 6), four showed differences between the sexes. Interestingly, all studies that examined cognitive outcomes reported sex differences. Functional independence or disability as an outcome post ABI reflected sex differences in eight out of 17 studies. Note, some studies examined functional independence in combination with motor and cognitive outcomes. See Table 1 for specific studies and outcomes.

#### Impact of neurorehabilitation technologies on functional outcomes

An initial search using the defined terms related to neurorehabilitation technologies' influence on functional outcome post-ABI returned 1,623 sources. 120 studies were analyzed beyond their title (see Figure 1). Studies that explicitly stated “robotics”, “virtual reality” or “brain-computer interface” alongside “traumatic brain injury”, “stroke”, or an analogous term such as “cerebral infarction”, were prioritized for a more thorough review. An additional search by hand was conducted to supplement knowledge on the impact of neurorehabilitation on TBI and cognitive outcomes specifically. The 21 studies that met eligibility criteria are included in Table 2 for their findings on neurorehabilitation technologies.

Most studies that examined the efficacy of neurorehabilitation technologies at improving functional outcomes post-ABI were studying stroke (*n* = 19). Upper extremity motor function was the most examined functional outcome and many of the functional outcome measures used were assessing motor function (e.g., FMA, BBS). It was more common for these studies to show mixed results when comparing the findings of multiple functional outcome measures, however, most studies did find the technology of interest to be efficacious in some capacity (*n* = 19). The most common type of technology that appeared in the initial search was robotic-assistance technologies or exoskeletons. There were a significant number of studies examining the efficacy of VR alone or in combination with other interventions. It is evident from the quantity and quality of literature available on brain computer interfaces that this technology is at a more preliminary stage when it comes to neurorehabilitation. See Table 2 for specific studies and outcomes.

#### Comparison of functional outcomes impacted by neurorehabilitation technologies and sex

**Figure 2:**
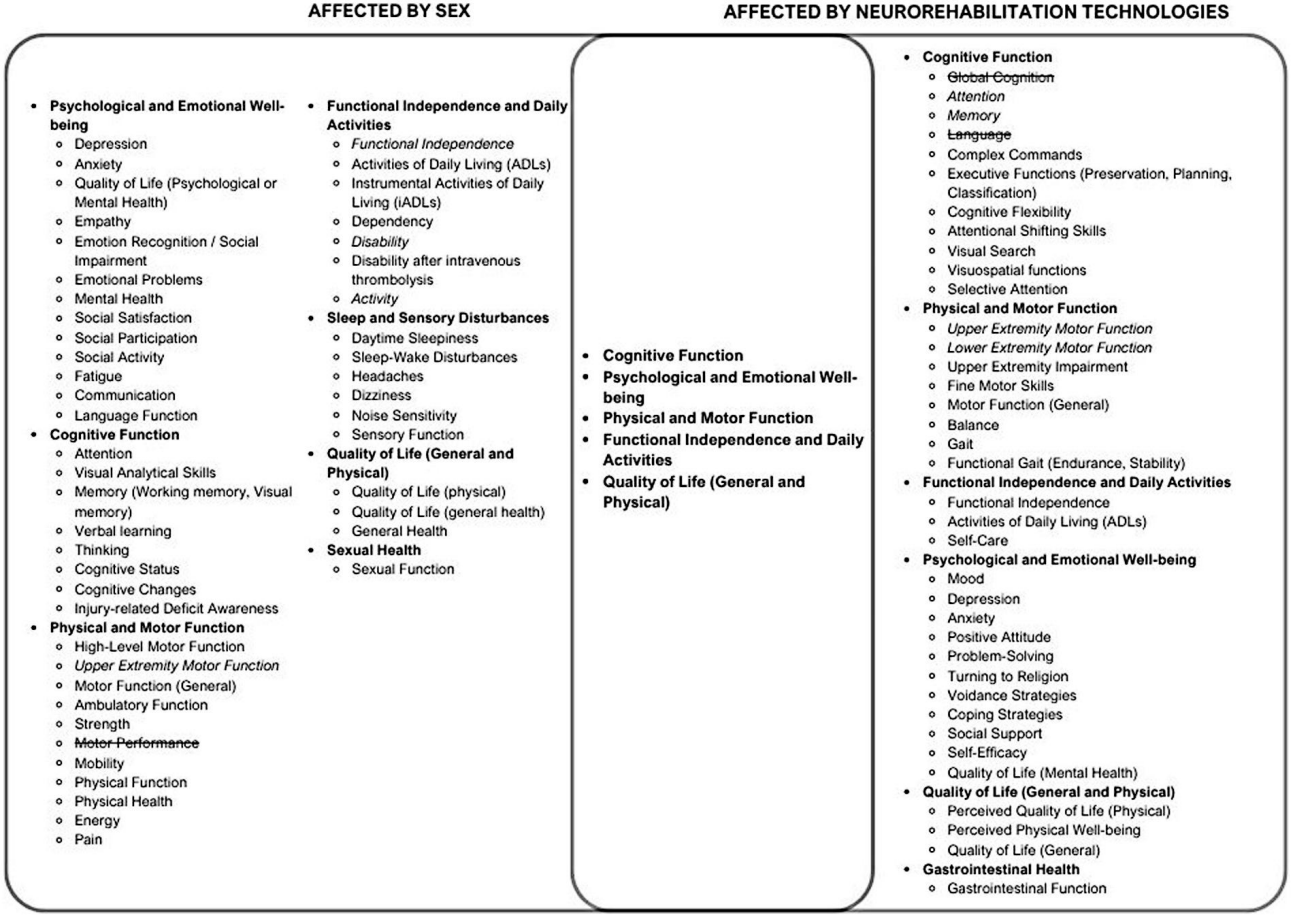
Venn diagram of functional outcomes proposed to be affected by neurorehabilitation technologies and Sex. Functional Outcomes found to be unaffected by sex or neurorehabilitation technologies, respectively, were represented with a strikethrough. Italicized functional outcomes had mixed results in terms of the effect of either sex or neurorehabilitation technologies. Although the Venn diagram provides a simplified visual overview of the conceptual overlap of the functional outcomes impacted by sex and neurorehabilitation technologies, it does not fully capture the complexity or contextual variability of the respective impacts, particularly due to the grouping of individual functional outcomes into more general domains. The representation is not intended to be a comprehensive model, and interpretations should consider these inherent limitations.

To understand how sex might influence the efficacy of neurorehabilitation technologies at improving functional outcomes post-ABI, a comparison of functional outcomes affected by sex and neurorehabilitation technologies, as identified in Tables 1, 2, was conducted. All functional outcomes mentioned in the analyzed studies were included in the comparison shown in Figure 2 and categorized into more general functional outcome domains (e.g., cognitive function, psychological and emotional well-being etc.). Of the eight general functional outcome domains analyzed, five domains were impacted by both sex and neurorehabilitation technologies, and three were impacted by sex or neurorehabilitation technologies alone.

In Figure 2, functional outcome domains found to be unaffected by sex or neurorehabilitation technologies, respectively, were represented with a strikethrough. Italicized functional outcomes had mixed results in terms of the effect of either sex or neurorehabilitation technologies. The general functional outcome domains that were affected by both sex and neurorehabilitation are present in the center of the Venn diagram and include cognitive function, psychological and emotional well-being, physical and motor function, functional independence and daily activities, and quality of life (general and physical). Although the Venn diagram provides a simplified visual overview of the conceptual overlap of the functional outcomes impacted by sex and neurorehabilitation technologies, it does not fully capture the complexity or contextual variability of the respective impacts, particularly due to the grouping of individual functional outcomes into more general domains. The representation is not intended to be a comprehensive model, and interpretations should consider these inherent limitations.

## Discussion

This narrative review indicates that sex may intersect with the efficacy of neurorehabilitation technologies in certain functional domains (notably cognitive and psychosocial outcomes), but not in others (such as general functional independence or upper extremity motor recovery). As such, this review provides a rationale that sex may only need consideration when studying the efficacy of neurorehabilitation technologies at improving certain functional outcomes post-ABI. However, there are a few considerations to note when interpreting these findings. For example, the sheer variety of measures used to evaluate functional outcomes in the reviewed literature, the lack of consensus across studies, and the limited availability of evidence for certain types of ABI and technologies.

### Measurement comparability

Previous research has shown that comparability of the findings across functional outcome scales is limited (90, 91, 108). When comparing self-reported (Barthel-20) and performance-based (TUG, 30s-CST, AMPS) functional outcome scales for elderly patients in the emergency department, Neilsen et al. (90) concluded that these scales reflect different aspects of a patient's functional ability. Additionally, Zdravkovic et al. (108) found the correlation between patient-reported outcome measures and clinician-reported outcome measures to be low to moderate. More specific to functional outcome scales used to evaluate ABI, Sato et al. (91), found the percentage of patients declared “independent” was significantly reduced using the FIM, in comparison to the mRS. Although this research speaks to the importance of using a wide variety of scales to assess functional recovery, it also calls for more rigorous research on the impacts of sex on the efficacy of neurorehabilitation technology. Not to mention, it denotes the methodology of comparing functional outcomes measured by different scales as a central methodological limitation affecting the conclusions of this review. Despite measurement limitations, the findings of this review revealed interesting insights on the intersection of sex and neurorehabilitation technology for certain functional domains.

### Functional domain insights

The most studied functional outcome across both sex and neurorehabilitation technology studies was functional independence, otherwise referred to as disability, or ADLs. These were most commonly measured by the FIM, mRS, or GOS-E. Interestingly, the studies that employed the FIM or GOS-E to examine sex-differences in functional outcomes post-ABI often found there to be no significant difference between the sexes (41, 52, 53, 57); Those employing the mRS found otherwise, although there were a few outliers (16, 17, 47–49, 65). FIM is generally found to have greater sensitivity in comparison to global scales of disability such as the mRS (91, 92). Given these findings, there is some evidence to suggest that sex may require less attention when developing neurorehabilitation technologies aimed at improving functional independence alone. That said, functional independence was not the primary or sole outcome of interest for any of the neurorehabilitation technology studies reviewed.

Due to the use of different functional outcome scales across the studies reviewed, the specific functional outcomes examined within the general function domains varied across sex and neurorehabilitation technology studies. For instance, although both sex and neurorehabilitation technologies were found to influence psychological and emotional wellbeing, sex was shown to have greater influence on social dimensions of this domain (39, 42, 56, 66). A couple of neurorehabilitation technology studies examined influence on social aspects, but these were listed under the general domain of psychological well-being (80, 82). Given that social factors have been considered a driving factor of sex-differences in response to ABI, they may be important to contemplate in future studies on the efficacy of neurorehabilitation technology studies (11, 12, 18).

In terms of the physical and motor functional domain, upper extremity motor function was by far the most studied functional outcome within the neurorehabilitation technology studies; this is likely due to weakness or paralysis in an upper limb being a common outcome post-stroke (109). Dahlby et al. (64) examined the impact of sex on recovery of upper extremity motor function post-stroke and found there to be no significant impact. Roth et al. (56) gives some evidence to sex impacting recovery of hand function post-stroke, but their research design was not as rigorous as Dahlby et al. The studies analyzed in this review do not provide sufficient rationale to consider sex when developing neurorehabilitation technologies aimed at improving upper extremity motor function. That said, this example only considers stroke, urging further discussion of any potential differences between stroke and TBI when considering the impact of sex on interventions within this review.

### ABI type considerations

The limited availability of studies examining the effects of neurorehabilitation technologies on functional recovery post-TBI specifically, impacts the ability to generalize the findings of this review across ABI (53, 68). Based on the findings of this review, it is more reasonable to suggest that the impacts of sex should be considered for stroke alone. However, in examining the modality by which neurorehabilitation technology works to improve functional outcomes, there is some rationale to including TBI in the findings (93). For instance, robotic-assistance technology works by inducing neuroplasticity through repetitive movement and biofeedback (25). Neuroplasticity is an important mechanism for recovery following both stroke and TBI, lending argument that findings related to neurorehabilitation technologies can be comparable between these types of ABI (93–96). Additionally, the potential for neuroplasticity may differ between the sexes, giving further evidence that the primary insult causing ABI may not be of utmost importance when determining the impact of sex on neurorehabilitation technology's efficacy (97–99). No less, it is important to recognize differences in injury-types. For example, studies examining the efficacy of VR at improving functional outcomes post-stroke found VR to be significantly effective at improving motor function but not cognitive outcomes (69–71). Alternatively, a study by De Luca et al. (28, 29) found VR to be effective at improving an abundance of cognitive functions (i.e., executive functions, visuospatial functions, and attentional shifting) post-TBI. The difference in these results highlights the importance of considering injury-specific factors.

To conclude, the findings of this review urge researchers to carefully consider methodological decisions when studying ABI interventions, including the selection of measurement tools and study population (e.g., ABI type), to ensure comparability and validity of results. Additionally, clinicians and researchers should likely consider sex when studying, developing or employing neurorehabilitation technology for a variety of functional outcomes (Figure 2), but there appears less rationale to do so when examining functional independence alone, or upper extremity motor function.

## Future research

As additional primary research on neurorehabilitation technologies becomes available, future research on how sex affects the efficacy of these technologies should involve a more systematic or direct approach (e.g., sex-stratified RCTs) and provide actionable recommendations on how findings should inform practice. Bannigan et al. (100), and von Groll et al. (101), head in the right direction by examining the direct impacts of sex on VR and BCI, respectively. Bannigan et al. find that sex contributes to differences in VR-induced motion sickness, and von Groll et al. build on existing research related to sex-differences in the ability to control motor imagery BCI. Placing findings such as these in the context of neurorehabilitation will strengthen the ability to inform practice.

Multiple stakeholders play a role in ensuring sex is appropriately considered in the development and use of neurorehabilitation technologies. Consideration of sex should begin with developers; this might include incorporating human-centered design principles into the development process, ultimately making the end user a central focus from inspiration to implementation and allowing for early acknowledgement of individual complexities (102). Researchers and clinicians also play a central role, for instance, future research that aims to explore equitable use of healthcare technologies should be mindful of confounding factors such as age, race, and socioeconomic status, and use subgroup meta-analysis and inclusive participant recruitment to address these factors. Gender as a social construct and non-binary individuals should also be considered in determining the influence of sex as we continue to strive towards more equitable and personalized technology (103, 104).

Based on the results of this review, further research is required to clarify the role of neurorehabilitation technologies in TBI. Additional investigations are needed to better understand how sex influences social functional outcomes, certain cognitive functions, sexual health, and sleep and sensory disturbances (Figure 2). Moreover, studies examining sex differences in gastrointestinal health post-ABI could provide further insights into the influence of sex on certain neurorehabilitation technologies that target this functional outcome (Figure 2).

## Limitations

As a narrative review, the findings presented here are inherently affected by the author's perspective and do not provide a conclusive understanding of the effect of sex on the efficacy of neurorehabilitation technologies post-ABI. The chosen methodology—comparison of heterogenous outcome measures, combination of multiple ABI-types, and inclusion of varied neurorehabilitation technologies—also affects the reliability and specificity of this review, as described in the discussion section. Further methodological limitations include the usage of a single non-blinded reviewer and non-systematic inclusion leading to potential selection bias. This review is limited by not thoroughly examining the impact of age and injury severity on sex-differences, given that sex hormones and their influence on functional recovery post-ABI are affected by ageing (105). The exclusion of non-English studies also limits international generalizability.

## Conclusion

Through the comparison of functional outcomes affected by sex with the functional outcomes affected by technology, this review aimed to determine if rationale exists for studying, developing, or employing neurorehabilitation technologies differently in the sexes. Analysis of 59 studies shows that the impact of sex on the efficacy of neurorehabilitation technologies is complex, varying according to the specific functional outcome targeted, the type of injury, and the measurement scale employed. In particular, there is some evidence to suggest that sex require less attention when employing technologies aimed solely at improving upper extremity motor function post-stroke or functional independence.

As neurorehabilitation technologies continue to evolve, and sex differences in functional recovery post-ABI are further elucidated, sex should remain an important consideration, particularly due to the potential impact of sex on the capacity for neuroplasticity, the mechanism underpinning these technologies (97–99). To the author's knowledge, no prior review has aligned with Health Canada's SGBA + to specifically examine sex influences on the efficacy of rehabilitation technologies in the context of neurorehabilitation, making this analysis a novel contribution.

## Data Availability

The original contributions presented in the study are included in the article/Supplementary Material, further inquiries can be directed to the corresponding author/s.

## Glossary

Acquired brain injury (ABI): An acquired brain injury is any injury to the brain that occurs after birth and is not the result of a genetic or developmental disorder. Traumatic brain injury and stroke are considered acquired brain injuries.

Augmented Reality: A technology that overlays digital information or graphics onto the user's view of the real world. Unlike virtual reality, which provides a fully simulated environment, augmented reality supplements the physical environment with virtual elements, often through devices such as smartphones or specialized glasses.

Brain Computer Interface: A technology that enables direct communication between the brain and an external device. By translating brain signals into commands, BCIs allow users to control computers, prosthetics, or other machines without traditional muscle-based inputs.

Cognitive Functional Recovery: The return of pre-injury ability levels for cognitive functions—including but not limited to speech, language processing, attention, visual-spatial processing, and memory—as measured by patient-reported functional outcomes or medical observation of functional outcomes.

Complete Functional Recovery: The complete return to previous activity levels following an injury or the absence of a disability originally caused by the injury.

Exoskeleton: A wearable, external mechanical structure designed to support or enhance a person's movement and strength. In rehabilitation settings, exoskeletons can assist individuals with impaired mobility to stand, walk, and perform other movements by providing additional mechanical force or stability.

Functional Outcomes: Various measures used to determine a person's recovery in different areas of life (social, physical, occupational, etc.) following injury. Functional outcomes differ from clinical outcomes in that they do not measure specific symptoms but rather the return of functional abilities.

Moderate to severe Traumatic brain injury (msTBI): An injury to the brain caused by blunt force or trauma resulting in a loss of consciousness for more than 30 min or coma.

Motor Functional Recovery: The return of pre-injury ability levels to perform voluntary movements and postures, as measured by patient-reported functional outcomes or medical observation of functional outcomes.

Neurogenesis: The process by which new neurons are formed in the brain. In humans, neurogenesis primarily occurs during embryonic development but continues in certain brain regions (such as the hippocampus) into adulthood, playing a role in learning, memory, and recovery after injury.

Post-Acute Rehabilitation Period: The period of 6–12 months following injury.

Robot-Assisted Gait Training: A therapeutic approach that employs robotic devices to help individuals learn or relearn how to walk, often after nervous system injury. The robot provides support and guides the lower limbs through a gait pattern, facilitating repetitive, task-specific practice.

Robot-Assisted Task-Oriented Training: A rehabilitation approach that uses robotic devices to aid in the repetitive practice of functional tasks relevant to daily living or therapy goals. This method integrates robots capable of providing assistance or resistance during movements, enabling patients to practice targeted tasks in a controlled, intensive manner. By focusing on functional activities, robot-assisted task-oriented training promotes motor learning and neural plasticity, often leading to improved motor function and independence in individuals with neurological impairments.

Stroke: An injury to the brain caused by a loss of blood flow due to blood clots or broken blood vessels in the brain.

Synaptic Transmission: The process by which one neuron communicates with another or with a target cell (such as a muscle or gland) across a synapse. This involves the release of neurotransmitters from the presynaptic neuron, their diffusion across the synaptic cleft, and their binding to receptors on the postsynaptic cell, thereby triggering or modulating its electrical or chemical response.

Synaptogenesis: The formation of new synapses (the points of contact where neurons communicate). Synaptogenesis occurs extensively during early brain development but can also take place throughout life in response to learning, experience, and certain types of rehabilitation.

Virtual Reality: A computer-generated simulation that immerses users in a three-dimensional, interactive environment. VR systems often use headsets and motion-tracking devices to provide a realistic sense of presence in a digital world, which can be employed for training, rehabilitation, and entertainment.
